# Surgical Emergencies in Rectal Cancer: A Narrative Review

**DOI:** 10.3390/jcm14010126

**Published:** 2024-12-29

**Authors:** Maria Pajola, Paola Fugazzola, Lorenzo Cobianchi, Simone Frassini, Ahmed Ghaly, Carlo Bianchi, Luca Ansaloni

**Affiliations:** 1 General Surgery Department, Fondazione IRCCS Policlinico San Matteo, 27100 Pavia, Italy; maria.pajola01@universitadipavia.it (M.P.); l.cobianchi@smatteo.pv.it (L.C.); simone.frassini01@universitadipavia.it (S.F.); ahmed.ghaly01@universitadipavia.it (A.G.); cm.bianchi@smatteo.pv.it (C.B.); l.ansaloni@smatteo.pv.it (L.A.); 2 PhD in Experimental Medicine, Department of Internal Medicine and Medical Therapy, University of Pavia, 27100 Pavia, Italy; 3 Department of Clinical, Diagnostic and Pediatric Sciences, University of Pavia, 27100 Pavia, Italy

**Keywords:** rectal cancer, emergency surgery, acute rectal bleeding, rectal perforation, obstructive rectal cancer

## Abstract

Colorectal cancer (CRC) is the third most diagnosed cancer worldwide and the second most common cause of cancer death. About 20% of patients diagnosed with rectal cancer present with emergency symptoms. Typical symptoms include acute bleeding, obstruction, and perforation. These emergency situations can be life-threatening and may lead to decreased life expectancy and quality of life. Bowel perforation is the most common cause of emergency presentation, followed by obstruction and acute bleeding. This narrative review analyzes the existing literature regarding the acute presentation of rectal cancer, producing three flow charts for the management of the main rectal emergencies. The treatment of acute bleeding differs based on the hemodynamic status. Treatment for bowel perforation or occlusion differs depending on whether the lesion is intraperitoneal or extraperitoneal. Emergency presentations seem to be strongly associated with several poor prognostic factors, including lymphovascular invasion, perineural invasion, and high-grade or poorly differentiated tumors. An association between emergency presentation and larger tumor size, advanced tumor stage, node-positive disease, and metastatic disease is reported in the literature. The difference between colon and rectal cancer, both in terms of treatment and prognosis, has been widely acknowledged. Thus, comprehensive studies and dedicated guidelines are needed, considering the lack of literature published about rectal cancer in an emergency setting.

## 1. Introduction

According to the International Agency for Research on Cancer, Colorectal cancer (CRC), which includes both colon and rectal cancer, is the third most diagnosed cancer worldwide, and it is the second most common cause of cancer death [1]. In the European Union, rectal cancer (RC) incidence is 125,000 cases per year, and the mortality rate is 4–10/100,000 deaths per year [2]. Even though the mortality rate has been decreasing [3], the 5-year survival rate for rectal cancer is 67% [4]. Differently from colon cancer, rectal cancer affects younger patients, and its annual incidence has increased by 2.1% in this group [5].

Risk factors for developing CRC are age, inflammatory bowel disease (ulcerative colitis, Crohn’s disease), a family history of CRC in first-degree relatives, smoking, lifestyle, and diet (such as a high intake of red meat and alcohol consumption [6], diabetes mellitus, metabolic syndrome, and high body mass index).

CRC incidence can be lowered by screening average-risk patients, as detecting and removing precancerous polyps and early-stage cancer can reduce mortality. Early diagnosis has a significant impact on survival [3]. Screening modalities differ according to nations’ organized programs; the latest National Comprehensive Cancer Network (NCC) guidelines consider the following to be feasible screening tests: the fecal occult blood test (FOBT), the fecal immunochemical test, the multitargeted stool DNA (mt-sDNA) test, colonoscopy, flexible sigmoidoscopy, and CT colonography [7,8,9,10,11].

Patients may present in a clinic or emergency room with rectal bleeding or blood in the stool or they may report changes in stool habits (diarrhea or constipation), significant weight loss, and fatigue. Physical examination with digital rectal examination may reveal a palpable tumor. Carcinoembryonic antigen (CEA) levels may be found to be elevated. A complete colonoscopy with biopsy for histopathological examination is crucial for the diagnosis of rectal cancer. After RC diagnosis, thorough disease staging with computed tomography (CT) of the chest and abdomen is mandatory. The most frequent sites of metastasis are the liver (20 to 30% of patients with CRC) and the lungs (4% to 9%) [12]. In patients with RC, defining the locoregional extension of the disease is necessary to select the most appropriate therapy: pelvic magnetic resonance (MRI) is the most accurate technique for this purpose. At the same time, Endoscopic Rectal Ultrasound may be used for the earliest lesions. Cancer patients’ management should be discussed among specialized and dedicated multidisciplinary teams (MDTs) [2,12].

The management options depend on the disease stage at diagnosis (early or advanced) and location (high or low rectum) [2,4,12]. It is essential to discriminate between intraperitoneal and extraperitoneal tumors. The difference in terms of treatment and prognosis is significant. High rectal neoplasms are often considered similar to colon neoplasms. On the other hand, neoplasms of the extraperitoneal rectum carry a higher risk of local recurrence (LR), which prompts the evaluation of a possible preoperative treatment.

Pre-invasive disease and early T1 rectal cancers in the absence of high-risk features and lymph node involvement can be treated with minimally invasive techniques like endoscopic submucosal dissection, endoscopic mucosal resection, transanal endoscopic microsurgery, and transanal minimally invasive surgery. After the procedure, close follow-up is pivotal for the patient’s survival.

Neoadjuvant chemoradiation (NACR) followed by surgery is the standard of care for patients with Locally Advanced Rectal Cancer (LARC). The primary surgical endpoint is complete tumor resection and lymphadenectomy. The surgical options are local excision, anterior rectal resection with sphincter-sparing procedures, and abdominoperineal resection with total mesorectal excision (TME). TME is the excision of all the mesorectal fat with the regional lymph node through meticulous dissection [13].

There are several distinct options for the NARC regimen when it comes to treating rectal cancer. Traditionally, the main strategies have involves either long-course chemoradiotherapy (LCRT) or short-course radiotherapy (SCRT). Recently, total neoadjuvant therapy (TNT) has emerged as a promising alternative. This approach involves completing all cycles of chemotherapy and radiotherapy before surgery, and it has received approval as an effective treatment option from both the European Society of Medical Oncology (ESMO) [2] and the National Comprehensive Cancer Network (NCCN) [12].

A recent meta-analysis conducted by Donnelly et al. [14], pooling data from 15 different studies, found that TNT significantly improves overall survival compared to both LCRT and SCRT. Additionally, it was shown to reduce overall recurrence rates when compared to LCRT. Moreover, this study showed no significant differences in disease-free survival, local recurrence rates, R0 resection rates, treatment toxicity, or patient compliance between TNT, SCRT, and LCRT.

This narrative review analyzes the published literature concerning the acute presentation of rectal cancer. We thoroughly examine acute rectal bleeding, obstruction, and perforation. We have included three flow charts outlining the management strategies for each of these emergency situations. These flow charts aim to facilitate better decision-making and prompt treatment in clinical practice, ensuring that patients receive the timely care they need during these critical emergencies.

## 2. Emergency Presentation

About 20% of patients diagnosed with colorectal cancer present with emergency symptoms. Typical symptoms include acute bleeding, obstruction, and perforation. These emergency situations can be life-threatening and may lead to decreased life expectancy and quality of life.

### 2.1. Acute Rectal Bleeding

Acute Lower Gastrointestinal Bleeding (LGIB) is one of the most common causes of emergency presentation in patients with rectal cancer. Rectal cancer can erode blood vessels, leading to active bleeding. The typical presentation is haematochezia, the presence of bright red blood with or without stool, usually with anemia. The worst-case scenario is hypovolemic shock in unstable patients.

The American College of Gastroenterology (ACG) reported an overall incidence of LGIB ranging between 33 and 87 patients out of 100,000 [15]. The leading cause of LIGB is diverticular bleeding [15], followed by anorectal disease such as hemorrhoidal bleeding. Less common causes are colorectal cancer, colitis, radiation proctitis, and iatrogenic bleeding. Different risk factors have been identified, such as advanced age [16], alcohol consumption, and smoking.

Upon patient presentation, a focused history of comorbidities and medications, physical examination—including digital rectal examination—and an assessment of hemodynamic parameters should be performed. Associated symptoms to investigate include abdominal pain, changes in bowel habits, and unintended weight loss. Around 75% of cases of LGIB are self-limiting and chronic. Chronic bleeding may be occult.

Patients presenting with bleeding should be assessed based on hemodynamics. Patients may be categorized as stable or unstable. Monitoring vital signs—blood pressure, heart rate, and oxygen saturation—is essential while ensuring intravenous access. The British Society of Gastroenterology (BSG) [17] suggests using the Shock Index (heart rate divided by systolic blood pressure) as a marker of active bleeding. The European Society of Gastrointestinal Endoscopy (ESGE) [18] guidelines advise against using a single score to determine whether to admit patients to the hospital. According to the ESGE and the British guidelines, discharge and outpatient evaluation may be considered when patients present with a self-limited bleed and an Oakland score of ≤8 points. The ACG considers it feasible for low-risk patients, identified through tools like the Oakland score or SHA2PE, to be managed conservatively and discharged with outpatient follow-up. This may reduce healthcare costs and resource use. Different prediction scores have been developed for LGIB, such as the NOBLADS and Birmingham scores, but their clinical use is limited. While promising, further validation studies are necessary before routine adoption in clinical practice. These tools supplement clinical judgment, but they do not replace it. In a survey with 52 institutions enrolled, the Society of Abdominal Radiology (SAR) [19] reported that less than 50% of those institutions use a specific algorithm to manage LGIB.

For hemodynamically stable patients, a colonoscopy during the hospital stay should be performed [18]. A systematic review and meta-analysis [20] of 21 studies pointed out that early colonoscopy does not lead to decreased rebleeding, mortality, or need for surgery. The review reported an incidence of rebleeding of 13.5% (95% CI, 11.8–15.5%) and an incidence of mortality related to acute LGIB of 1.1% (95% CI, 0.6–1.8%), with no significant difference between patients undergoing early or late colonoscopy. An increase in the identification of bleeding sources and a decrease in the length of hospitalization was noted with the performance of early colonoscopy.

**Figure 1:**
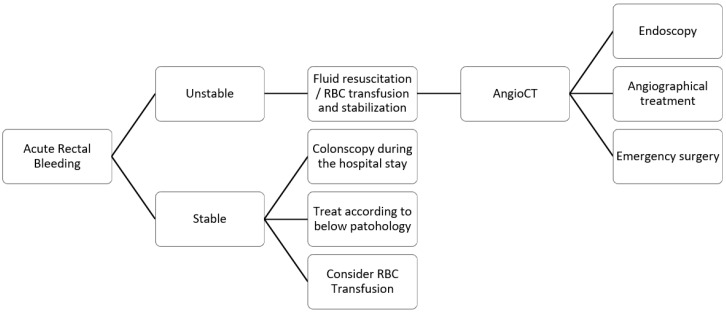
Flowchart for management of rectal bleeding (AngioCT, Angio-Computed Tomography; RBC, Red Blood Cell).

According to the last guidelines published by the ESGE [18], hemodynamically unstable patients should undergo a CT scan before endoscopy or angiographical treatment to localize the source of bleeding (Figure 1). A meta-analysis [21] pooling data from twenty-two studies assessed the accuracy of CT scans, showing an overall sensitivity of 85.2% (95% CI: 75.5% to 91.5%) and an overall specificity of 92.1% (95% CI: 76.7% to 97.7%). Endoscopic clipping, endoscopic band ligation, and the local administration of epinephrine are options for the endoscopist to control the bleeding. Transcatheter arterial embolization is a new treatment option that is feasible only for patients with life-threatening conditions. Another meta-analysis [22] also reported embolization as the most effective procedure for diverticular bleeding.

Emergency surgery should be performed only when patients with severe bleeding cannot be stabilized with endoscopy or angiography procedures. The advantages are removing the source of bleeding and, in the case of cancer, the tumor. However, emergency rectal resection can be challenging, with the risk of a suboptimal lymphadenectomy and a higher risk of colostomy.

In a nationwide UK audit conducted in 2015 [23], the proportion of LGIB attributed to polyps and colon cancer was lower than 1%. Furthermore, the audit found that only 2.3% of patients were in hemodynamic shock, and only 0.2% underwent surgery. A prospective population-based study [24] states that colonoscopy found colorectal cancer in 12% of cases; moreover, they found that patients with CRC have clinically significant bleeding.

Radiotherapy could be effective for bleeding control in palliative settings. A single-center study [25] confirmed that palliative radiotherapy could control gastrointestinal bleeding in 89% of patients, with an overall re-bleeding rate of 25%. Shorter regimens were also suggested considering short life expectancy and palliative settings.

### 2.2. Obstructive Rectal Cancer

Obstructive rectal cancer occurs in 8% to 29% of patients with CRC [26]. Patients with obstructive rectal cancer may present with abdominal pain, the absence of flatus, and stool with abdominal distension. Other frequent findings are changes in bowel habits, vomiting, nausea [27], and a lack of bowel sounds. Digital Rectal Exploration can be used to diagnose lower rectal cancer. Abdominal X-rays may show dilated loops and air–fluid levels. A CT scan with intravenous contrast is the gold standard imaging technique to evaluate bowel obstruction for the diagnostic work-up. Considering that the tumors that cause obstruction are usually locally advanced, a CT scan also allows for an assessment of the stage of the lesion.

Fluid resuscitation, electrolyte correction, and NG tube placement are the first steps in managing these patients, while emergency colonoscopy is usually not indicated.

However, bowel obstruction must be treated promptly to prevent progression to ischemia or perforation. Several treatment options exist, including self-expandable metal stents (SEMSs), loop colostomy, and proctectomy with or without anastomosis. When determining the best approach, it is recommended to consider the effectiveness of neoadjuvant therapy for patients with locally advanced rectal cancer. For this reason, the obstruction should be resolved as much as possible without removing the tumor, allowing the patient to undergo neoadjuvant therapy and subsequent elective surgery [28], offering the best therapeutic approach to optimizing the oncologic outcomes.

**Figure 2:**
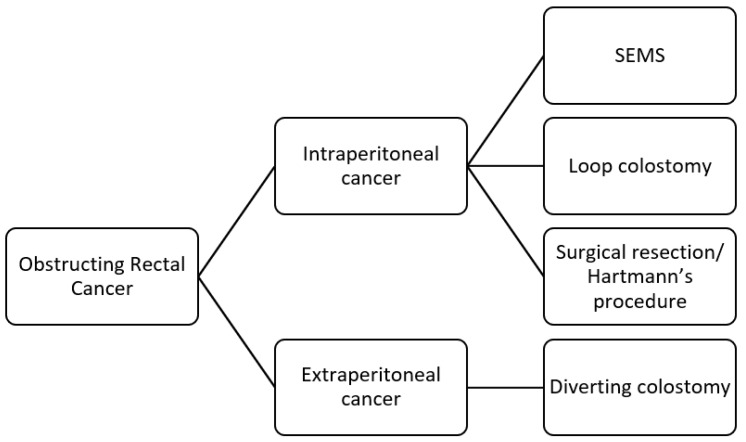
Flowchart for management of obstructive rectal cancer (SEMS: self-expandable metal stent).

To define the appropriate management, it is crucial to assess the distance of the tumor from the anal verge (Figure 2). Patients with proximal lesions may benefit from endoscopic metal stents such as SEMSs, the creation of a diverting stoma, or surgical resection of the tumor. SEMSs can be introduced through a colonoscope, and it was initially used for palliative treatment. Today, a SEMS can serve as a “bridge to surgery”, resolving the obstruction without immediate surgical intervention [29]. This approach allows for optimizing the patient’s condition, tumor staging, possible neoadjuvant therapy, and the opportunity for elective surgery. A recent systematic review and meta-analysis [30] found a technical success rate for this approach of 97% but a clinical success rate of only 69%, with an overall peri-procedure complication rate of 28%. Another meta-analysis [31], pooling data from seventeen studies, demonstrated that long-term oncological outcomes are comparable between patients treated with SEMS as a bridge to surgery and those undergoing emergency surgery. However, while SEMS has been associated with increased long-term complications, such as delayed perforation, stent obstruction or migration, chronic pain, and local recurrence, it is associated with a lower rate of stoma formation. SEMS is also preferred over colostomy for palliation [28,32,33].

Loop colostomy allows surgeons to address bowel obstruction without performing rectal resection. Its advantages include a shorter procedure time and the possibility of administering neoadjuvant therapy. Conversely, a rectal resection with anastomosis or Hartmann’s procedure offers oncological resection of rectal cancer. Still, it comes with increased risks, such as suboptimal lymphadenectomy and, in the case of Hartmann’s procedure, the fashioning of a permanent stoma [34]. According to the latest WSES guidelines, in cases of obstructive intraperitoneal rectal cancer, resection with primary anastomosis should be chosen over Hartmann’s procedure in the absence of other risk factors. Nonetheless, Hartmann’s procedure remains the most performed surgery for colon surgical emergencies worldwide.

The management strategies for obstructing extraperitoneal rectal cancer are different from those used for intraperitoneal cases. The latest WSES guidelines [28] recommend against primary resection to facilitate neoadjuvant therapy. A stoma should be created, with a transverse colostomy being the preferred option. The placement of a self-expanding metal stent (SEMS) is generally not advised for these patients, as it may not offer adequate relief [28]. Therefore, the surgical approach should focus on stabilizing the patient while preserving options for further oncological treatment and optimizing outcomes.

### 2.3. Rectal Perforation

Rectal perforation has a variety of causes, such as cancer (36%), spontaneous perforation (20%), iatrogenic perforation (20%), and diverticulitis (19%) [35]. The incidence of perforation due to cancer ranges from 3% to 10% [36]; the mortality rate of secondary peritonitis after perforation is 30–50%.

Usually, patients are admitted to the Emergency Room with fever, tachypnoea, and confusion with a diffuse or localized tender abdomen; rebound may be present. Laboratory tests usually show leukocytosis, neutrophilia, elevated amylase levels, and lactic acidosis [28]. With a clinical suspicion of bowel perforation, an abdominal plain X-ray is usually the first level of imaging requested, but it has suboptimal sensitivity. A CT scan can confirm the diagnosis, with a sensitivity of 95–98% and specificity of 95–97% [37], and can identify synchronous metastasis. Common findings are free air near the site of bowel perforation [38], abscess or focal collection of extramural fluid, bowel segments with wall thickening, and pneumatosis [39].

Rectal perforation has two typical presentations. Firstly, it may present as a free perforation with diffuse peritonitis caused by the intraluminal tumor growth, which leads to obstruction and then to distal perforation. The result is more frequently a free perforation with stool leakage and diffuse peritonitis. The second presentation is a peritumoral abscess or tumor necrosis. These findings are likely due to a perforation at the primary tumor site, resulting in more localized peritonitis. Emergency surgery is the standard of care for CRC perforation. The priority is to control sepsis in patients with diffuse peritonitis. Broad-spectrum antibiotics should be initiated immediately, and source control must be obtained. The tumor should be resected when feasible. According to the latest WSES guidelines [28], oncological resection should be performed. In cases of significant peritoneal contamination or poor bowel quality, an ostomy should be considered to avoid complications from primary anastomosis. If the perforation occurs at the tumor site, bowel resection with or without anastomosis and fashioning of an ostomy are indicated. When the perforation is proximal to the tumor, the lesion and the site of proximal perforation should be managed through resection.

**Figure 3:**
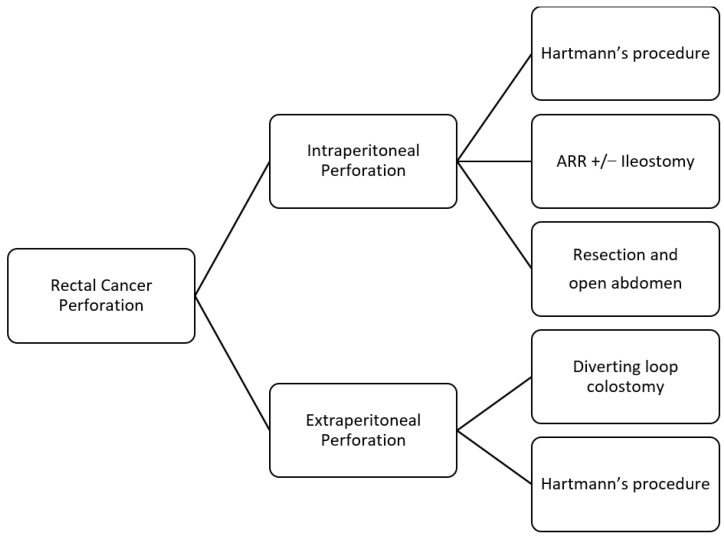
Flowchart for the management of rectal cancer perforation (ARR: Anterior Rectal Resection).

In emergency settings, treatment approaches differ based on whether the lesion is intraperitoneal or extraperitoneal. Intraperitoneal lesions can be treated similarly to perforation due to colon cancer. Emergency surgery is required; rectal resection with primary anastomosis or Hartmann’s procedure and colostomy creation are the possible treatments, and the treatment is chosen based on the patient’s condition. Anterior rectal resection (ARR) with primary anastomosis and, eventually, with a temporary diverting ileostomy may be considered in selected hemodynamically stable patients with upper rectal perforation cases. For unstable patients, bowel resection with an open abdomen may be valuable (Figure 3).

Perforated extraperitoneal rectal cancer may present as an acute anorectal abscess [40] or fistula (rectovaginal or rectovesical fistulas); its presentation and diagnosis can be challenging. Cancer could be suspected in patients with atypical findings such as perineal sepsis, a long history of persistent perianal disease, and advanced age [41]. Abscess formation is rare and occurs in 0.3 to 0.4% of CRC patients. The treatment of pelvic abscesses is undefined: antibiotics and surgery are valid options. Hartmann’s procedure or diverting loop colostomy are valid options for extraperitoneal lesions. US-guided or CT-guided percutaneous drainage can be a possibility, but considering the risk of spreading cell cancer, they should preferably be avoided.

Rectal cancer that presents as a perianal abscess progressing to Fournier’s gangrene (FG) is a rare phenomenon described in the literature [42,43]. The treatment of FG involves extensive debridement of the areas of necrosis in association with broad-spectrum antibiotics. There are no guidelines or consensus on the timing of the resection of primary locally advanced cancer [44].

Rectal perforation following radiotherapy for rectal cancer is a rare but severe complication. During radiotherapy, high doses of radiation are targeted at the cancerous tissue to destroy malignant cells. However, this treatment can also cause unintended damage to surrounding healthy tissues, including the rectal wall. Perforation is a medical emergency that requires immediate attention. Treatment often requires surgical intervention to obtain source control. A retrospective study [35] by Shinkawa et al. showed that associated septic shock, diffuse peritonitis, and concurrent end-stage renal failure were all significant risk factors for early postoperative mortality. The mortality related to perforation was around 30–40%.

## 3. Discussion

Colorectal cancer (CRC) is one of the most diagnosed cancers worldwide. CRC often has its first presentation as a clinical and surgical emergency. Up to 20% of diagnosed patients require emergency surgery; this is a pivotal challenge in clinical practice, influencing both short-term and long-term patient outcomes.

Implementing national screening programs is an effective strategy to reduce emergency presentations; adherence to such programs may lead to earlier detection of potential issues [8]. A meta-analysis by Bretthauer et al. [11] studied whether cancer screening tests are associated with increased life expectancy; their study included four different types of cancer (CRC and lung, prostate, and breast cancer) and six common screening tests (mammography, colonoscopy, sigmoidoscopy, FOBT, computed tomography, and prostate-specific antigens). Out of the six tests examined, only sigmoidoscopy significantly extended both survival and longevity in patients with colorectal cancer (CRC).

Bowel perforation is the most common cause of emergency presentation in colorectal cancer patients, occurring in 8% to 60% of cases [45]. It is followed by obstruction and acute bleeding. Bowel perforation, obstruction, and acute bleeding may require emergency surgical procedures to manage immediate life-threatening complications. These procedures, while potentially life-saving, frequently carry a greater risk of morbidity and mortality compared to elective surgeries. Higher morbidity and mortality seem to be mainly due to the advanced stage of disease at presentation and the acute nature of the complication.

A large population-based study [46] reported that up to 12% of rectal cancer patients were first admitted as emergencies. This study underscores the burden that emergency presentations have on the healthcare system. Moreover, older age, smoking habits, and pre-existing comorbidities were reported as common factors among those admitted as emergencies. These findings emphasize the importance of early detection and intervention, particularly in high-risk groups, to prevent emergencies.

A social issue was also reported: public patients may have more limited access to endoscopic exams than private-sector ones. This disparity can delay diagnosis and treatment, but addressing this issue could lead to earlier diagnoses and fewer emergencies.

Emergency presentations are more frequently observed in patients with advanced-stage colorectal cancer [47]. A systematic review and meta-analysis by Golder et al. [48] examined 54 studies to explore the association between clinicopathological factors and emergency presentations. Their findings established that emergency presentations were strongly associated with several poor prognostic factors, including lymphovascular invasion, perineural invasion, and high-grade or poorly differentiated tumors. Their meta-analysis also reported an association between emergency presentation and larger tumor size (*p* = 0.011), advanced tumor stage (*p* < 0.001), node-positive disease (*p* < 0.001), and metastatic disease (*p* < 0.001). The findings of their meta-analysis emphasize the aggressive nature of tumors presenting as emergencies, which may lead to the poorer outcomes observed in this group.

A study by Talebreza et al. [49] involving 67 patients reported similar conclusions, showing that patients’ mortality was closely linked to the TNM stage (*p* = 0.026) and tumor grade (*p* = 0.02). The higher the stage and grade, the worse the prognosis, particularly in those requiring emergency surgery. The outcomes following emergency surgery are worse than those for elective procedures [49,50]. A systematic review by Zamaray et al., including data from eleven retrospective cohort studies [51], reported that patients with free perforation have significantly worse clinical outcomes. This study highlights different emergency presentations (free perforation or contained perforation) as indicators of short-term mortality following a colorectal cancer diagnosis. Similar findings emerged from a retrospective study that analyzed data from cancer patient residents in England, drawn from the National Cancer Data Repository (NCDR) [47].

The rising incidence of rectal cancer among younger patients is an emerging concern that may result in an increase in emergency presentations due to cancer lesions within this population. Essential questions about whether differences in tumor histopathology, stage, and age influence the likelihood of such presentations require extensive research to investigate these differences and their effects on clinical and oncological outcomes.

Neoadjuvant therapy followed by surgery is the standard of care for LARC patients. Patients with a complete clinical response (cCR) or a near-complete response (nCR) after neoadjuvant therapy may be considered for rectum-sparing approaches, such as a “watch and wait” strategy or local excision. These options are not standard of care but are becoming more widely accepted as effective treatment options. Nevertheless, comprehensive studies on long-term complications and emergency-related situations have not yet been published. Future research is needed to assess how these treatment strategies affect the rates and outcomes of emergency presentations

Minimally invasive surgical techniques, such as laparoscopic and robotic surgery, are increasingly being adopted in elective procedures. Different studies have shown that laparoscopic surgery is associated with similar short-term and long-term outcomes compared to traditional open surgery [52].

A recent meta-analysis [53], pooling data from randomized controlled trials, reported no significant differences between laparoscopic surgery and open surgery concerning local recurrence, distant recurrence, overall survival, and disease-free survival. Consequently, the authors concluded that laparoscopic surgery is non-inferior to open surgery regarding pathological and long-term oncological outcomes.

Recent advancements in robotics and artificial intelligence (AI) have significantly influenced the field of surgery. The integration of robotic systems allows for reducing recovery times and complications. A recent meta-analysis by Abdelsamad [54] compared short- and long-term outcomes between robot-assisted and laparoscopic mesorectal excision. The authors reported that the laparoscopic group was associated with higher overall recurrence than the robotic group, with an Odds Ratio of 2.02 (95% CI, 1.02–4.02, *p* = 0.049).

According to the latest NCCN guidelines, minimally invasive resection is not recommended for acute bowel obstruction or perforation [12]. However, laparoscopic surgery is becoming increasingly accepted in emergency settings. The application of robotic surgery in emergencies remains to be explored. Further research and analysis and even more widespread use of robotic surgery are needed to provide information on the potential advantages of this procedure.

## 4. Conclusions

Emergency presentations in rectal cancer remain a critical area of concern, affecting patients’ clinical outcomes.

Acute bleeding, bowel obstruction, and perforation—common emergency scenarios—require distinct management approaches. The effective management of acute bleeding relies on assessing the patient’s hemodynamic status. Conservative measures may be considered in patients with stable vital signs; hemodynamic instability requires more aggressive interventions and procedures. The management of bowel perforation or occlusion must consider the location of the lesion—intraperitoneal or extraperitoneal. This distinction is crucial in determining the correct surgical approach; morbidity and mortality differ depending upon lesion location.

Emergency presentations seem to be strongly associated with several poor prognostic factors, including lymphovascular invasion, perineural invasion, and high-grade or poorly differentiated tumors [48]. The published literature points to an association between emergency presentation and adverse tumor characteristics, such as increased size, advanced tumor stage, node-positive disease, and metastatic disease [49].

## 5. Future Directions and Limitations

The literature available on colorectal cancer emergencies is extensive; however, there is a lack of specificity regarding rectal cancer emergencies. Most of the published literature groups colon and rectal cancer together, which may overlook complications that are specifically related to rectal cancer. In recent years, researchers have begun to recognize and investigate the differences between these two types of cancer. However, there is still a significant gap in the literature. Specific guidelines on rectal cancer emergencies are yet to be published. Improving patient care and outcomes in acute settings may require the development of specific guidelines for the emergency management of rectal cancer and targeted research. Existing guidelines on colorectal cancer emergencies will provide a valuable and broad framework.

The limitations of this study include potential subjectivity in data interpretation. Furthermore, while the published literature was thoroughly reviewed and considered, there may have been insufficient systematization in selecting sources.
